# Estriol‐mediated neuroprotection in multiple sclerosis localized by voxel‐based morphometry

**DOI:** 10.1002/brb3.1086

**Published:** 2018-08-24

**Authors:** Allan MacKenzie‐Graham, Jenny Brook, Florian Kurth, Yuichiro Itoh, Cassandra Meyer, Michael J. Montag, He‐Jing Wang, Robert Elashoff, Rhonda R. Voskuhl

**Affiliations:** ^1^ Department of Neurology Ahmanson‐Lovelace Brain Mapping Center David Geffen School of Medicine at UCLA Los Angeles California; ^2^ UCLA Multiple Sclerosis Program Department of Neurology David Geffen School of Medicine at UCLA Los Angeles California; ^3^ Department of Biomathematics David Geffen School of Medicine at UCLA Los Angeles California

**Keywords:** atrophy, brain, estriol, magnetic resonance imaging, multiple sclerosis, voxel‐based morphometry

## Abstract

**Introduction:**

Progressive gray matter (GM) atrophy is a hallmark of multiple sclerosis (MS). Cognitive impairment has been observed in 40%–70% of MS patients and has been linked to GM atrophy. In a phase 2 trial of estriol treatment in women with relapsing–remitting MS (RRMS), higher estriol levels correlated with greater improvement on the paced auditory serial addition test (PASAT) and imaging revealed sparing of localized GM in estriol‐treated compared to placebo‐treated patients. To better understand the significance of this GM sparing, the current study explored the relationships between the GM sparing and traditional MRI measures and clinical outcomes.

**Methods:**

Sixty‐two estriol‐ and forty‐nine placebo‐treated RRMS patients underwent clinical evaluations and brain MRI. Voxel‐based morphometry (VBM) was used to evaluate voxelwise GM sparing from high‐resolution T1‐weighted scans.

**Results:**

A region of treatment‐induced sparing (TIS) was defined as the areas where GM was spared in estriol‐ as compared to placebo‐treated groups, localized primarily within the frontal and parietal cortices. We observed that TIS volume was directly correlated with improvement on the PASAT. Next, a longitudinal cognitive disability‐specific atlas (DSA) was defined by correlating voxelwise GM volumes with PASAT scores, that is, areas where less GM correlated with less improvement in PASAT scores. Finally, overlap between the TIS and the longitudinal cognitive DSA revealed a specific region of cortical GM that was preserved in estriol‐treated subjects that was associated with better performance on the PASAT.

**Conclusions:**

Discovery of this region of overlap was biology driven, not based on an *a priori* structure of interest. It included the medial frontal cortex, an area previously implicated in problem solving and attention. These findings indicate that localized GM sparing during estriol treatment was associated with improvement in cognitive testing, suggesting a clinically relevant, disability‐specific biomarker for clinical trials of candidate neuroprotective treatments in MS.

## 1 INTRODUCTION

Cognitive impairment is a commonly observed symptom in multiple sclerosis (MS), affecting 40%–70% of patients (Amato, Zipoli, & Portaccio, 2006; Chiaravalloti & DeLuca, 2008; Rao, Leo, Bernardin, & Unverzagt, 1991). It impacts patients personally, professionally, and socially, disrupting their lives and diminishing their quality of life. Cognitive impairment can be present early in disease, but is more common later, in the progressive forms of MS (Amato et al., 2006; Chiaravalloti & DeLuca, 2008). It can affect many systems, including attention, executive function, and long‐term memory; however, the most commonly affected domain in MS is processing speed (Amato et al., 2006; Chiaravalloti & DeLuca, 2008).

Progressive brain atrophy is another well‐known feature of MS and is considered a marker of irreversible tissue damage (Chard et al., 2002; Charil et al., 2007; Pirko, Lucchinetti, Sriram, & Bakshi, 2007). Quantitative magnetic resonance imaging (MRI) studies have demonstrated that gray matter (GM) atrophy occurs even in the earliest stages of disease (Calabrese, Atzori, et al., 2007; Dalton et al., 2004; De Stefano et al., 2003; Valsasina et al., 2005), develops faster than white matter (WM) atrophy (Chard et al., 2004; Fisher, Lee, Nakamura, & Rudick, 2008), and is strongly correlated with physical disability and cognitive impairment (Amato et al., 2004; Chard et al., 2002; Dalton et al., 2004; De Stefano et al., 2003; Sailer et al., 2003; Sanfilipo, Benedict, Sharma, Weinstock‐Guttman, & Bakshi, 2005; Sanfilipo, Benedict, Weinstock‐Guttman, & Bakshi, 2006). This appears to be true in both relapsing–remitting multiple sclerosis (RRMS) and benign MS, suggesting a silent progression of cognitive impairment independent of MS clinical course (Gonzalez‐Rosa et al., 2006). Indeed, while MS is characterized by WM lesions, GM atrophy is considered to be a relevant biomarker of permanent disability in MS (Bermel & Bakshi, 2006; van den Elskamp et al., 2010; Fisher et al., 2002). In fact, GM atrophy has been found to be associated with changes in all cognitive domains in MS (Morgen et al., 2006). This is not surprising as several pathological (Kutzelnigg et al., 2005; Peterson, Bo, Mork, Chang, & Trapp, 2001) and MRI (Calabrese, Atzori, et al., 2007; Calabrese, De Stefano, et al., 2007; De Stefano et al., 2003) studies have shown that the cerebral cortex is profoundly affected in MS. Indeed, cortical atrophy has been proposed as one of the major underlying substrates of cognitive impairment in MS (Amato et al., 2004; Benedict et al., 2004; Tekok‐Kilic et al., 2007). Which regions within the cerebral cortex undergo atrophy and align with impairments in which cognitive domains is only beginning to be investigated (Wen et al., 2015).

Current disease‐modifying treatments (DMTs) that target immune mechanisms have shown mixed results in preventing brain atrophy (Branger, Parienti, Sormani, & Defer, 2016). Some studies have reported no significant, or conflicting, effects of DMTs on brain atrophy (Bermel & Bakshi, 2006; Calabrese et al., 2012; Lublin et al., 2016; Tiberio et al., 2005). Others have reported statistically significant slowing of brain atrophy, but only after 24 months or more (Cohen et al., 2012; Coles et al., 2012; Kappos et al., 2010; Mattioli, Stampatori, Bellomi, Scarpazza, & Capra, 2015; Miller et al., 2007; Rinaldi et al., 2012). Brain atrophy is considered predictive of progression in cognitive and/or motor disability (Bermel & Bakshi, 2006; Deloire et al., 2011; Morgen et al., 2006), emphasizing a need for early intervention to prevent brain atrophy and forestall the advance of disability. Thus, fast‐acting directly neuroprotective treatments are needed to halt GM atrophy.

Voxel‐based morphometry (VBM) is a well‐established image analysis technique (Ashburner & Friston, 2001; Bookstein, 2001; Davatzikos, 2004; Friston & Ashburner, 2004) that can provide an unbiased and comprehensive assessment of anatomic differences throughout the brain. Indeed, VBM has been used extensively in the analysis of brain atrophy in MS (Battaglini et al., 2009; Bendfeldt et al., 2009, 2010; Ceccarelli et al., 2008; Raz et al., 2010). We have used VBM to demonstrate that there were distinct associations between voxelwise GM loss and specific clinical disabilities (MacKenzie‐Graham et al., 2016). Disability in the paced auditory serial addition test (PASAT) correlated with decreased GM in the primary auditory and premotor cortices, disability in the 9‐hole peg test correlated with decreased GM in the left inferior frontal gyrus (a region involved with fine motor control), and disability on the bowel and bladder functional system subscale of the extended disability status scale correlated with decreased GM in the right paracentral lobulus (a region known to be involved in micturition).

Only a few studies have attempted to use longitudinal VBM analyses to evaluate the effects of DMTs in MS clinical trials. One study demonstrated localized GM loss after 24 months of treatment with interferon beta (1a or 1b), but no comparison with placebo treatment was shown (Bendfeldt et al., 2010). Another study reported small regions of increased GM density in patients treated with natalizumab (Mattioli et al., 2015). Thus, the MS community is beginning to investigate the effect of treatment on localized GM loss at the voxel level. We hypothesize that determining the effects of treatment on localized GM atrophy using this unbiased, biology‐driven, VBM approach may lead to neuroprotective treatments optimized for specific brain regions and specific clinical disabilities. To this end, in a recently completed phase 2 clinical trial, we demonstrated that estriol treatment led to the preservation of localized GM compared to placebo in patients with MS and that higher levels of estriol in the blood were correlated with better performance on the PASAT (Voskuhl et al., 2016). Here, we investigate relationships between estriol treatment‐induced localized GM preservation and traditional MRI and clinical disability measures.

## 2 METHODS

### 2.1 Participants

Subjects for this study were enrolled as part of the phase 2 clinical trial entitled “A Combination Trial of Copaxone plus Estriol in RRMS” (trial identifier #NCT00451204). Eligibility criteria for the trial were that patients be female, 18–50 years of age, have a diagnosis of RRMS as defined according to the McDonald criteria (Polman et al., 2005) with a baseline score of 0–4.5 on the EDSS (Kurtzke, 1983), and have active disease defined by at least two documented relapses in the previous 24 months before screening or at least one documented relapse within 24 months before screening with a history of at least one gadolinium‐enhancing lesion on a brain or spinal cord MRI performed at least 3 months prior to or 3 months after the clinical relapse. Exclusion criteria were progressive forms of MS (Lublin & Reingold, 1996), other clinically significant diseases, exposure to glatiramer acetate for longer than 2 months prior to randomization, relapse or steroid use within 30 days of randomization, use of any interferon, adrenocorticotropic hormone, corticosteroids, intravenous immunoglobulins or other DMTs within 2 months prior to randomization, prespecified laboratory test abnormalities, those who are pregnant, breast‐feeding, or trying to get pregnant, those who have undergone surgical or natural menopause for longer than 1 or 3 years, respectively, with no hormone replacement therapy, those not willing to discontinue other hormonal treatments, and those who have ever been treated with major immunosuppressive contraindicated treatments.

The study was approved by each site's committee for the Protection of Human Research Subjects with written informed consent at trial screening. All patients provided their own glatiramer acetate (GA). GA was administered at 20 mg per day subcutaneously, and estriol (or placebo) was administered at 8 mg per day orally.

**Figure 1:**
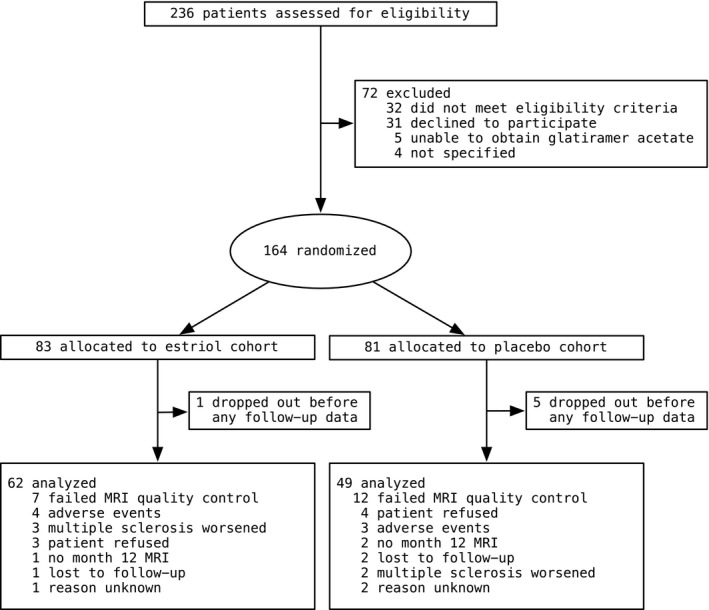
Trial profile

The subjects were enrolled at 16 sites throughout the USA. Patients underwent a neurologic examination, comprehensive neurologic, cognitive, and behavioral testing including the extended disability status scale, the MS functional composite, comprising the nine‐hole peg test (9HPT), the timed 25‐foot walk (T25FW), and the paced auditory serial addition test at 2 s (PASAT2), the paced auditory serial addition test at 3 s (PASAT3), and brain MRI (Table 1). Within a site, at least one estriol + GA and one placebo + GA subject needed to be enrolled and all subjects needed to be scanned on the same scanner with consistent parameters, so that potential differences by site could be estimated and accounted for in the statistical model (Supporting Information Table S1). For this study, all included subjects were required to have at least reached month 12 of the study and all images had to pass stringent, labor‐intensive visual quality control measures by an examiner blinded to treatment (FK). The images had to exhibit sufficient white/gray contrast and meaningful tissue segmentation, as well as a lack of artifacts and noise to be included in the analysis. Using these criteria, this study consisted of 111 subjects (62 in the estriol + GA group and 49 in the placebo + GA group) from 13 sites for month 12 analyses (Figure 1). Patients received either 8 mg oral estriol once per day (estriol + GA group) or placebo (placebo + GA group).

**Table 1:** Baseline characteristics

Baseline characteristics	All patients (*n* = 111)	Estriol + GA (*n* = 62)	Placebo + GA (*n* = 49)	*p* Value
Age (years)				
Mean ± *SD*	37.3 ± 7.5	37.4 ± 7.9	37.2 ± 7.1	0.72
Median, IQR	37.7, 32.7–43.2	39.0, 32.7–43.0	36.2, 32.6–43.8	
Range	20.0–53.6	20.0–53.6	20.6–51.0	
Ethnicity				
Black	11 (9.9%)	8 (12.9%)	3 (6.1%)	0.36^a^
Caucasian	90 (81.1%)	48 (77.4%)	42 (85.7%)	
Hispanic	9 (8.1%)	6 (9.7%)	3 (6.1%)	
Other	1 (0.9%)	0 (0.0%)	1 (2.0%)	
Education				
No college graduation	45 (40.5%)	27 (43.6%)	18 (36.7%)	0.47^a^
College graduation or higher	66 (59.5%)	35 (56.4%)	31 (63.3%)	
MS duration (years)				
Mean ± *SD*	3.1 ± 4.5	3.2 ± 4.3	2.9 ± 4.7	0.92
Median, IQR	0.7, 0.3–4.3	0.85, 0.3–5.1	0.6, 0.4–3.6	
Range	0.1–24.3	0.1–16.4	0.1–24.3	
Gd‐enhancing lesion volume (ml)				
Mean ± *SD*	65.1 ± 186.9	88.8 ± 236.7	35.1 ± 85.0	0.37
Median, IQR	0, 0–31.50	0, 0–50	0, 0–25	
Range	0–1162.8	0–1162.8	0–403	
FLAIR lesion volume (ml)				
Mean ± *SD*	5.7 ± 7.2	6.0 ± 6.9	5.7 ± 7.7	0.39
Median, IQR	3.1, 1.2–6.6	3.7, 1.4–7.0	2.7, 1.1–6.0	
Range	0.1–34.6	0.2–34.6	0.1–32.7	
GM volume (ml)				
Mean ± *SD*	587.8 ± 56.1	577.8 ± 55.4	600.5 ± 54.9	0.03
Median, IQR	584.1, 550.9–626.5	575.4, 548.6–611.6	595.2, 567.0–641.3	
Range	436.3–727.7	436.3–710.1	478.0–727.7	
WM volume (ml)				
Mean ± *SD*	493.4 ± 65.5	493.2 ± 61.7	493.5 ± 70.7	0.99
Median, IQR	486, 445.3–531.5	485.4, 456.5–524.4	494.8, 436.6–532.6	
Range	358.6–692.7	358.6–688.4	364.9–692.7	
CSF volume (ml)				
Mean ± *SD*	234.4 ± 48.9	231 ± 50.4	238.7 ± 47.1	0.23
Median, IQR	221, 200.2–254.8	219.8, 197.6–249.7	226.1, 210.3–258.7	
Range	161.6–380.1	161.6–374	163.2–380.1	
Cortical GM volume (ml)				
Mean ± *SD*	760.6 ± 40.7	755.6 ± 44.6	766.9 ± 34.6	0.16
Median, IQR	761.7, 731.9–795.9	755.3, 724.1–783.9	765.2, 747.7–801.3	
Range	660.0–849.8	660.0–849.8	684.4–823.1	
EDSS score				
Mean ± *SD*	2.2 ± 1.1	2.3 ± 1.1	2.1 ± 1.1	0.29
Median, IQR	2.0, 1.5–3	2.5, 1.5–3.0	2.0, 1.5–3.0	
Range	0–5.5	0–4.0	0–5.5	
MSFC Score				
Mean ± *SD*	0.15 ± 0.73	−0.09 ± 0.73	0.16 ± 0.74	0.05
Median, IQR	0.11, −0.48–0.55	0.048, −0.61–0.39	0.33, −0.21–0.77	
Range	−1.92–1.33	−1.92–1.26	−1.57–1.33	
PASAT3 score				
Mean ± *SD*	51.4 ± 9.1	50.3 ± 9.3	53.1 ± 8.6	0.07
Median, IQR	56, 46–59	54, 44–58	56, 50–59	
Range	26–60	26–60	27–59	
PASAT2 score				
Mean ± *SD*	41.8 ± 11.9	40.3 ± 12.3	43.7 ± 11.4	0.15
Median, IQR	43, 31–53	41, 29–51	45.5, 37–54	
Range	18–60	18–60	21–59	
9HPT				
Mean ± *SD*	19.4 ± 3.5	19.8 ± 4.1	19.0 ± 2.7	0.24
Median, IQR	18.8, 17.3–20.7	19.0, 17.6–21.0	18.7, 17.3–20.6	
Range	14.6–39.5	14.6–39.5	14.9–28.1	
T25FW				
Mean ± *SD*	4.81 ± 1.21	4.94 ± 1.13	4.65 ± 1.29	0.21
Median, IQR	4.65, 4.10–5.50	4.75, 4.15–5.60	4.45, 3.80–5.00	
Range	2.7–10.4	3.1–9.2	2.7–10.4	

*Notes*

CSF, cerebrospinal fluid; EDSS, expanded disability status scale; FLAIR, fluid‐attenuated inversion recovery; GA, glatiramer acetate; Gd, gadolinium; GM, gray matter; IQR, interquartile range; MSFC, multiple sclerosis functional composite; PASAT2, paced auditory serial addition test at 2 s; PASAT3, paced auditory serial addition test at 3 s; SD, standard deviation; T25FW, timed 25‐foot walk; WM, white matter; 9HPT, 9‐hole peg test.

a Chi‐square test; Wilcoxon's rank‐sum test for all others.

Imaging data used for this analysis were collected at the David Geffen School of Medicine at the University of California, Los Angeles, in Los Angeles, CA; the Washington University School of Medicine in St. Louis, MO; the University of Chicago Medical Center in Chicago, IL; the Wexner Medical Center at the Ohio State University in Columbus, OH; the Salt Lake City VA Medical Center in Salt Lake City, UT; the University of Texas Southwestern in Dallas, TX; the University of Colorado Denver in Aurora, CO; the University of Minnesota in Minneapolis, MN; the University of New Mexico Health Sciences Center in Albuquerque, NM; the University of Pennsylvania Perelman School of Medicine in Philadelphia, PA; the Johns Hopkins University, Baltimore, MD; the University of Kansas Medical Center in Kansas City, KS; and the Geisel School of Medicine, Dartmouth College in Hanover, NH.

A prespecified exploratory outcome measure was the PASAT. Practice tests were performed three times during screening, and alternative versions were used. Study assessments were performed at months 0 (estriol + GA *n* = 82, placebo + GA *n* = 76), 3 (estriol + GA *n* = 81, placebo + GA *n* = 75), 6 (estriol + GA *n* = 78, placebo + GA *n* = 63), 12 (estriol + GA *n* = 70, placebo + GA *n* = 63), 18 (estriol + GA *n* = 64, placebo + GA *n* = 57), and 24 (estriol + GA *n* = 60, placebo + GA *n* = 56). A linear mixed‐effects model was used to compare treatment groups at 12 months and 24 months. To estimate the difference in PASAT score change between the two study groups over all time points, baseline PASAT scores were used as an interaction term with treatment and month in the model. All patients’ follow‐up data over all 24 months were included (Voskuhl et al., 2016).

### 2.2 Image acquisition

Magnetic resonance imaging was acquired at 13 sites using T1‐weighted sequences with and without gadolinium‐based contrast agent, as well as a fluid‐attenuated inversion recovery (FLAIR) sequence to determine WM lesions. MRI was performed at 0, 3, 6, 12, and 24 months using a standardized protocol. Imaging parameters for each site are detailed in Supporting Information Table S1. MRI data were anonymized and then uploaded to the UCLA database. Before the study onset, each site provided a dummy scan utilizing the standardized sequences for review by UCLA investigators to verify scan quality and fidelity. Quality control was maintained at each site using standard procedures for clinical scanners (daily phantoms, stability testing). Phantoms were collected from 12 of the 15 sites quarterly, most using the standard American College of Radiology (ACR) phantom.

Images were reviewed locally by a radiologist blind to study details to assess for any new or unusual findings as a safety measure. Incoming imaging data were reviewed for completeness and fidelity to study pulse sequences by UCLA investigators. Local radiologists and imaging core investigators were all blind to randomization assignment. All MRI investigators remained blinded to treatment assignment until the end of the study.

### 2.3 Image analysis

Analysis of gadolinium‐enhancing lesions and FLAIR lesions was performed as described (Sicotte et al., 2002). Briefly, MRI data were coded by study site and randomization number. The number and volume of enhancing lesions were quantified on the postcontrast T1‐weighted scans using a semiautomated threshold‐based algorithm by a trained, experienced investigator (MM) who was blind to treatment group and verified by Dr. Nancy Sicotte. FLAIR images were inhomogeneity corrected, intensity‐normalized, and registered into a common space defined by the baseline image for each individual. All subsequent images were registered to the baseline image for spatial normalization using a rigid body transformation. FLAIR lesion volumes were determined using a semiautomated, intensity‐based segmentation procedure by an experienced researcher (MM) and verified by Dr. Nancy Sicotte.

Cortical GM volumes were determined using pairwise Jacobian integration (PJI) as described (Voskuhl et al., 2016).

Voxel‐based morphometry analyses were performed as previously described (Kurth et al., 2014). Brain images were preprocessed utilizing Statistical Parametric Mapping 8 (SPM8; http://www.fil.ion.ucl.ac.uk/spm/software/spm8) and the VBM8 toolbox (http://www.neuro.uni-jena.de/vbm) as previously described (Kurth et al., 2014; Luders, Gaser, Narr, & Toga, 2009). White matter lesions were in‐painted to minimize their impact (Ceccarelli et al., 2012; Chard, Jackson, Miller, & Wheeler‐Kingshott, 2010) based on manual delineations that were used for the analysis of new T2 lesions. The manually delineated lesion masks were coregistered to the T1‐weighted images, corrected if necessary, and used for lesion in‐painting as described by Chard et al. (2010). The lesion in‐painted images were subsequently realigned for each subject using halfway registrations and corrected for bias‐field inhomogeneities. The realigned, bias corrected images were then tissue‐classified into GM, WM, and cerebrospinal fluid (CSF) and registered to Montreal Neurological Institute (MNI) space through linear and nonlinear transformations (Ashburner, 2007; Kurth et al., 2014; Luders et al., 2009) (see http://dbm.neuro.uni-jena.de/vbm8/VBM8-Manual.pdf). More specifically, the tissue classification was based on maximum a posteriori segmentations (Rajapakse, Giedd, & Rapoport, 1997), accounted for partial volume effects (Tohka, Zijdenbos, & Evans, 2004), and was refined by applying a spatially adaptive nonlocal means denoising filter (Manjon, Coupe, Marti‐Bonmati, Collins, & Robles, 2010) as well as a hidden Markov random field model (Cuadra, Cammoun, Butz, Cuisenaire, & Thiran, 2005). These methods made the tissue classification independent of tissue probability maps and thus additionally minimized the influence of misclassifications, lesions, and altered geometry (Ceccarelli et al., 2012). Diffeomorphic Anatomical Registration using Exponentiated Lie Algebra (DARTEL) (Ashburner, 2007) was used to spatially normalize the GM segments to the DARTEL template supplied with the VBM8 toolbox (see http://dbm.neuro.uni-jena.de/vbm), resulting in a voxelwise comparability between subjects and time points. The spatially normalized and modulated GM, WM, and CSF compartments were used to calculate GM, WM, and CSF volumes. Finally, the GM segments were smoothed with a Gaussian kernel (8 mm full width at half maximum). These smoothed GM segments constituted the input for the statistical analysis. In order to visualize the relation between significant findings and the underlying mean anatomy of the sample, a mean template was created from the normalized brain images of all subjects.

### 2.4 Treatment‐induced sparing

**Figure 2:**
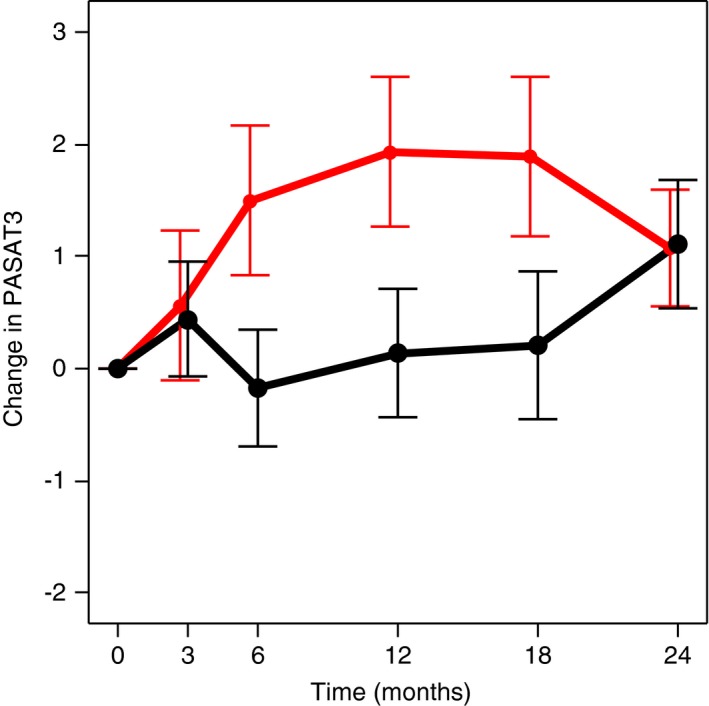
Change in paced auditory serial addition test at 3 s (PASAT3) scores in all patients at baseline month 0 and on treatment at months 3, 6, 12, 18, and 24. Change in mean absolute scores at each time point as compared to baseline is shown in red for the estriol + glatiramer acetate group and in black for the placebo + glatiramer acetate group. Values are expressed as means ± standard error of the mean. Positive change in absolute scores from baseline indicates improvement

The region of treatment‐induced sparing (TIS) was defined as the collection of clusters of GM that were significantly preserved in estriol + GA‐treated, but not in placebo + GA‐treated, subjects after 12 months of treatment (Figure 2) (Table 2) (Voskuhl et al., 2016). We observed clusters of preserved GM in the estriol + GA‐treated subjects, but none in the placebo +GA‐treated subjects. For the statistical analysis, a general linear model was applied that used the smoothed GM segments as the dependent variable and group × time as the independent variable. Subject and scan site were added as variables of no interest, thus effectively controlling for interindividual differences (e.g., individual anatomy, age, and disease duration) as well as the potentially confounding impact of different scanners. Nonsphericity was modeled and accounted for as described previously and implemented in SPM8 (Glaser & Friston, 2007). Applying this model, we calculated the interaction between group and time using *t*‐tests to investigate group differences in local GM changes between month 0 and month 12. In addition, the GM loss within each group was investigated by calculating *t*‐tests for month 0 > month 12 for each group separately. All results were corrected for multiple comparisons by controlling the false discovery rate (FDR) (Hochberg & Benjamini, 1990) using a threshold of *p* ≤ 0.05.

**Table 2:** Treatment‐induced sparing. Anatomic location of areas of reduced gray matter loss in estriol + glatiramer acetate (GA) cohort compared to placebo + GA cohort after 12 months of treatment

Anatomic structure	MNI coordinates			Cluster size (voxels)	Cluster size (ml)	*Z* score
	*X*	*Y*	*Z*			
Left frontal cortex	−22	14	48	3,382	11.4	4.68
Medial parietal cortex	4	−7	64	2,203	7.4	4.61
Left parietal cortex	−44	−46	46	1,817	6.1	4.12
Medial frontal cortex	−2	38	36	1,393	4.7	4.43
Left parietal cortex	−60	−22	31	731	2.5	4.23
Right temporal cortex	56	0	9	642	2.2	4.29
Medial parietal cortex	4	−51	12	490	1.7	4.16
Medial occipital cortex	3	−64	−3	297	1.0	4.19

*Note*

MNI, coordinates (*x*,* y*,* z*) according to Montreal Neurological Institute space.

### 2.5 Disability‐specific atlas

Correlations with PASAT2 were assessed using a general linear model with the preprocessed GM volumes as the dependent variable and the PASAT2 scores, treatment group, time, scanner, and subject as the independent variables. This removed the potentially confounding variances associated with treatment group, time, scanner, and interindividual variability, thus allowing an assessment of the partial correlations between local GM volume and PASAT2 scores. Again, nonsphericity was modeled and accounted for as described (Glaser & Friston, 2007), and results were corrected for multiple comparisons by controlling the FDR (Hochberg & Benjamini, 1990) using a threshold of *p* ≤ 0.05.

### 2.6 Statistical analyses

Baseline characteristics were presented as means with standard deviations or frequencies with percentage. The differences in demographics between the two groups were measured using Wilcoxon's test and chi‐square test, respectively. To compare estriol and placebo, absolute difference between baseline and 12 months in the outcome variables was calculated for all participants as well as by group. Heat map and correlation clustering were created using the “gplots” package in R (http://www.R-project.org). Differences of change in these outcomes between groups were assessed using linear regression analyses and Pearson's correlation coefficient. Due to the relatively small sample size in this exploratory study, the alpha level was only adjusted for multiple comparisons in the voxelwise analyses, not in the whole GM analyses. All analyses were carried out using SAS/STST software version 9.4.

## 3 RESULTS

In the published phase 2 trial, estriol + GA as compared to placebo + GA treatment improved cognitive performance in analyses including all time points (*p* = 0.038, between groups) (Voskuhl et al., 2016). As the placebo + GA group took the PASAT at the same time points as the estriol + GA group, this between‐group difference was not due to a practice effect. PASAT scores at each of the time points (months 0, 3, 6, 12, 18, and 24) are shown in Figure 2. Effects of estriol + GA treatment compared to placebo + GA began at month 6, persisted to month 12 and to month 18, but were lost at month 24. Poor compliance in both the estriol + GA and placebo + GA groups, with decreased estriol blood levels in the estriol + GA group, was observed at month 24, end of the trial (16.2 ng/ml at month 3 compared to 10.2 ng/ml at month 24, *p* = 0.003), coinciding with loss of improvement in cognitive performance at this single month 24 time point. When analyzing all time points, cognitive improvement correlated with higher estriol blood levels (*p* = 0.030) (Voskuhl et al., 2016). As this underscored the need for maintenance of estriol blood levels for continued benefit on cognitive performance, we focused our in‐depth MRI analysis herein on change from baseline to month 12, instead of month 24. Subgroup analysis previously showed greater PASAT improvement in estriol + GA‐treated subjects with impairment at baseline (PASAT3 scores less than 55) than those with no impairment (PASAT3 scores 55–60) (Voskuhl et al., 2016); however, we included all subjects in the analyses herein.

**Figure 3:**
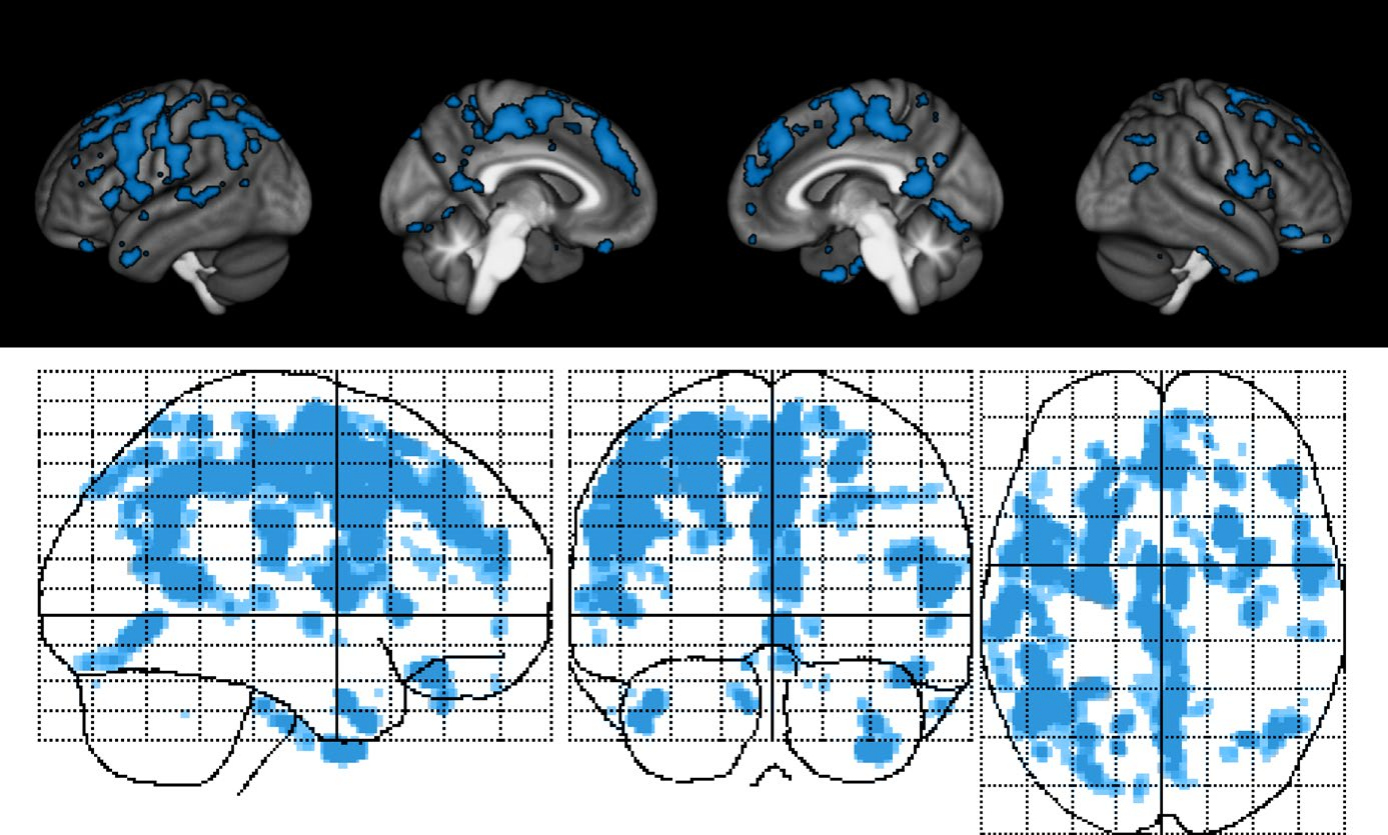
Estriol treatment‐induced sparing of gray matter. Surface renderings (top) and maximum intensity projections (bottom) of regions of significant gray matter preservation in the estriol + glatiramer acetate group as compared to the placebo + glatiramer acetate group at month 12 superimposed on the statistical parametric mapping standard glass brain. Results were corrected for multiple comparisons by controlling the false discovery rate at *p* ≤ 0.05

Voxel‐based morphometry analysis of GM sparing unveiled a region of TIS, a collection of predominantly cortical clusters of GM that was significantly preserved in estriol + GA‐treated subjects, but not in placebo + GA‐treated subjects, after 12 months of treatment (Figure 3) (Table 2) (Voskuhl et al., 2016). The analyses were performed bidirectionally, and we did not observe any clusters that exhibited sparing in the placebo +GA‐treated subjects compared to the estriol + GA‐treated subjects. Interestingly, while the study was not powered to reach significance for this exploratory MRI measure, there was a mean difference of 3.01 ± 0.69% in TIS GM volume (95% CI 1.65–4.37, *p* < 0.0001) between the estriol + GA cohort and the placebo + GA cohort at month 12. Further analysis demonstrated that GM preservation in the TIS persisted to month 24 (estriol + GA *n* = 46, placebo + GA *n* = 44), a mean difference of 1.57 ± 0.73% in TIS volume (95% CI 0.13–3.00, *p* = 0.03), despite significantly decreased blood estriol levels at the end of trial, month 24, in estriol‐treated patients (Voskuhl et al., 2016).

Regarding traditional MRI measures, we observed less annualized whole GM loss in the estriol + GA‐treated group (0.5%) than in the placebo + GA‐treated group (1.5%) (*p* = 0.04) when we evaluated the longitudinal changes from month 0 to month 12 (Table 3). When we focused our attention on the cerebral cortex, we observed a trend toward cortical GM sparing in the estriol + GA group compared to the placebo + GA group (*p* = 0.06). We also observed an improvement on PASAT3 performance in the estriol + GA group compared to the placebo + GA group (*p* = 0.02) and a trend toward improvement on the PASAT2 in the estriol + GA group compared to the placebo + GA group (*p* = 0.07). No differences were observed at baseline in gadolinium‐enhancing lesions, FLAIR lesion volume, whole brain volume, WM volume, cortical GM volume, nor voxelwise GM volume (by VBM) between the estriol + GA‐treated subjects and the placebo + GA‐treated subjects, with the exception that the estriol + GA‐treated subjects had a smaller GM volume than the placebo + GA‐treated subjects at baseline (*p* = 0.03).

**Table 3:** Mean longitudinal absolute change in clinical and MRI measurements between baseline and month 12

Specific clinical measurements	All (111)	Estriol + GA (62)	Placebo + GA (49)	*p* Value
Treatment‐induced sparing volume	−0.28	0.06	−0.70	<0.0001
Whole GM volume	−5.68	−2.95	−9.09	0.04
Cortical GM volume	−3.99	−2.94	−5.36	0.06
WM volume	−0.51	−1.42	0.63	0.59
CSF volume	3.31	1.48	5.61	0.04
Gd‐enhancing lesion volume	−0.05	−0.06	−0.03	0.22
FLAIR lesion volume	1.30	1.50	1.06	0.44
PASAT3	1.26	2.27	−0.02	0.02
PASAT2	1.17	2.07	0.00	0.07
9HPT	−0.49	−0.55	−0.42	0.70
T25FW	0.00	0.04	−0.05	0.61
MSFC	0.13	0.16	0.09	0.36
EDSS	−0.18	−0.19	−0.19	0.99

*Note*

CSF, cerebrospinal fluid; EDSS, expanded disability status scale; FLAIR, fluid‐attenuated inversion recovery; GA, glatiramer acetate; Gd, gadolinium; GM, gray matter; MRI, magnetic resonance imaging; MSFC, multiple sclerosis functional composite; PASAT2, paced auditory serial addition test at 2 s; PASAT3, paced auditory serial addition test at 3 s; T25FW, timed 25‐foot walk; WM, white matter; 9HPT, 9‐hole peg test.

**Figure 4:**
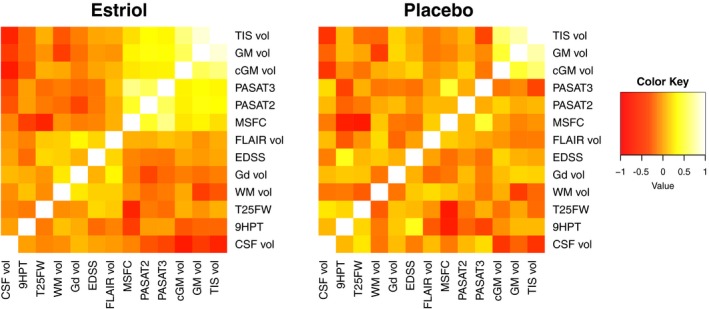
Heat map showing positive and negative correlations and their clustering in each treatment group. A graphical representation of relationship among treatment‐induced sparing volume (TIS vol), traditional MRI measures (gray matter volume (GM vol), cortical gray matter volume (cGM vol), fluid‐attenuated inversion recovery lesion volume (FLAIR vol), gadolinium‐enhancing lesion volume (Gd vol), white matter volume (WM vol), cerebrospinal fluid volume (CSF vol)), and clinical measures (paced auditory serial addition test at 3 s (PASAT3), paced auditory serial addition test at 2 s (PASAT2), timed 25‐foot walk (T25FW), nine‐hole peg test (9HPT), multiple sclerosis functional composite (MSFC), expended disability status score (EDSS)). In the heat map, yellow indicates a positive (direct) correlation and red indicates a negative (indirect) correlation between the values. (a) Heat map for the estriol + glatiramer acetate group. (b) Heat map for the placebo + glatiramer acetate group

We were especially interested in the relationship between GM sparing in the TIS and changes in traditional MRI measures and other clinical outcome measures; therefore, regression analyses were performed among the various measures. When displayed as heat maps, the estriol + GA group demonstrated a pattern of strong association between TIS volume, whole GM volume, cortical GM volume, PASAT3 scores, and PASAT2 scores (Figure 4a). In contrast, the heat map of the placebo + GA group did not show this pattern (Figure 4b). Neither group showed significant associations between TIS volume and gadolinium‐enhancing lesion volume or FLAIR lesion volume nor between TIS volume and EDSS, 9HPT, or T25FW.

Pearson's correlation coefficients and significance levels between TIS volumes and both MRI and clinical measures were then calculated. TIS volume directly correlated with better performance on both the PASAT3 and PASAT2 in the estriol‐treated, but not the placebo‐treated, group. In the estriol + GA group, TIS volume directly correlated with whole and cortical GM and inversely correlated with WM and CSF (Table 4).

**Table 4:** Pearson's correlation coefficients between absolute change in treatment‐induced sparing volume and the absolute change in MRI and clinical measurements

Specific clinical measurements	All (111)	Estriol + GA (62)	Placebo + GA (49)
Whole GM volume	0.84***	0.89***	0.77***
Cortical GM volume	0.53***	0.54***	0.46***
WM volume	−0.23 *	−0.27*	−0.21
CSF volume	−0.59***	−0.65***	−0.51***
Gd‐enhancing lesion volume	−0.03	−0.07	0.11
FLAIR lesion volume	0.03	0.04	−0.13
PASAT3	0.20*	0.31*	−0.34*
PASAT2	0.31**	0.34**	0.08
9HPT	−0.09	−0.21	0.09
T25FW	−0.01	0.06	−0.19
MSFC	0.15	0.22	−0.06
EDSS	0.02	0.02	0.03

Notes

CSF, cerebrospinal fluid; EDSS, expanded disability status scale; FLAIR, fluid‐attenuated inversion recovery; GA, glatiramer acetate; Gd, gadolinium; GM, gray matter; MRI, magnetic resonance imaging; MSFC, multiple sclerosis functional composite; PASAT2, paced auditory serial addition test at 2 s; PASAT3, paced auditory serial addition test at 3 s; T25FW, timed 25‐foot walk; WM, white matter; 9HPT, 9‐hole peg test.

*p ≤ 0.05, **p ≤ 0.01, ***p ≤ 0.001.

**Figure 5:**
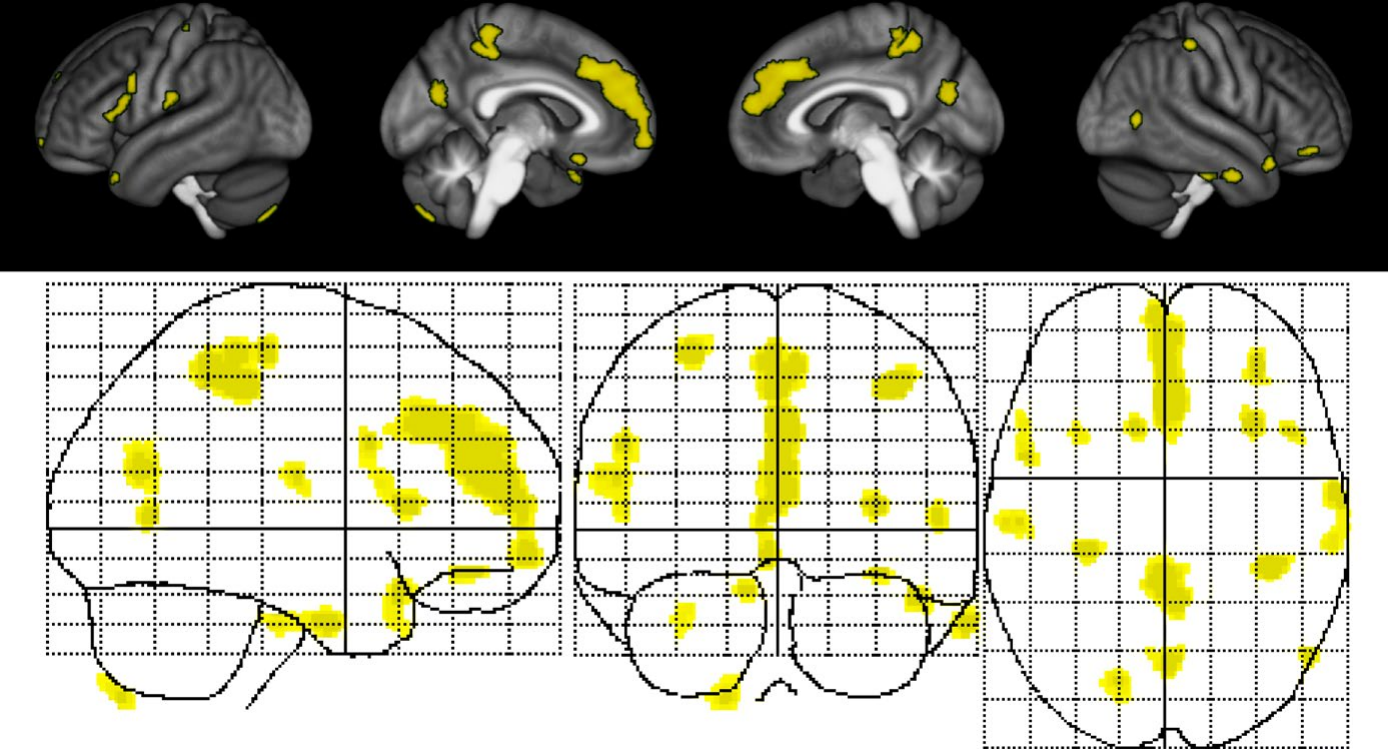
Longitudinal cognitive disability‐specific atlas. Surface renderings (top) and maximum intensity projections (bottom) of regions of statistically significant correlation between gray matter volume change and paced auditory serial addition test at 2‐s performance change in all patients and across all time points superimposed on the statistical parametric mapping standard glass brain. All results were corrected for multiple comparisons by controlling the false discovery rate at *p* ≤ 0.05

In a previous *cross‐sectional* approach using only the baseline month 0 data from this trial, we demonstrated that specific disabilities in MS patients were associated with lower voxelwise GM volumes in clinically eloquent regions. We observed that worse performance on the PASAT2 correlated with less GM in both auditory and premotor cortices (MacKenzie‐Graham et al., 2016). A broader continuum of disability on PASAT2 compared to PASAT3 provided a larger domain for this voxelwise regression analysis (MacKenzie‐Graham et al., 2016). For this study, we used a *longitudinal* approach to define a disability‐specific atlas (DSA) for the PASAT2 to reveal GM changes that correlate with PASAT2 performance. We defined this longitudinal cognitive DSA as the region where the change in GM volume correlated with the change in PASAT score, using all patients and all time points (Figure 5). Specifically, it is where GM preservation correlated with improvement in PASAT scores.

**Figure 6:**
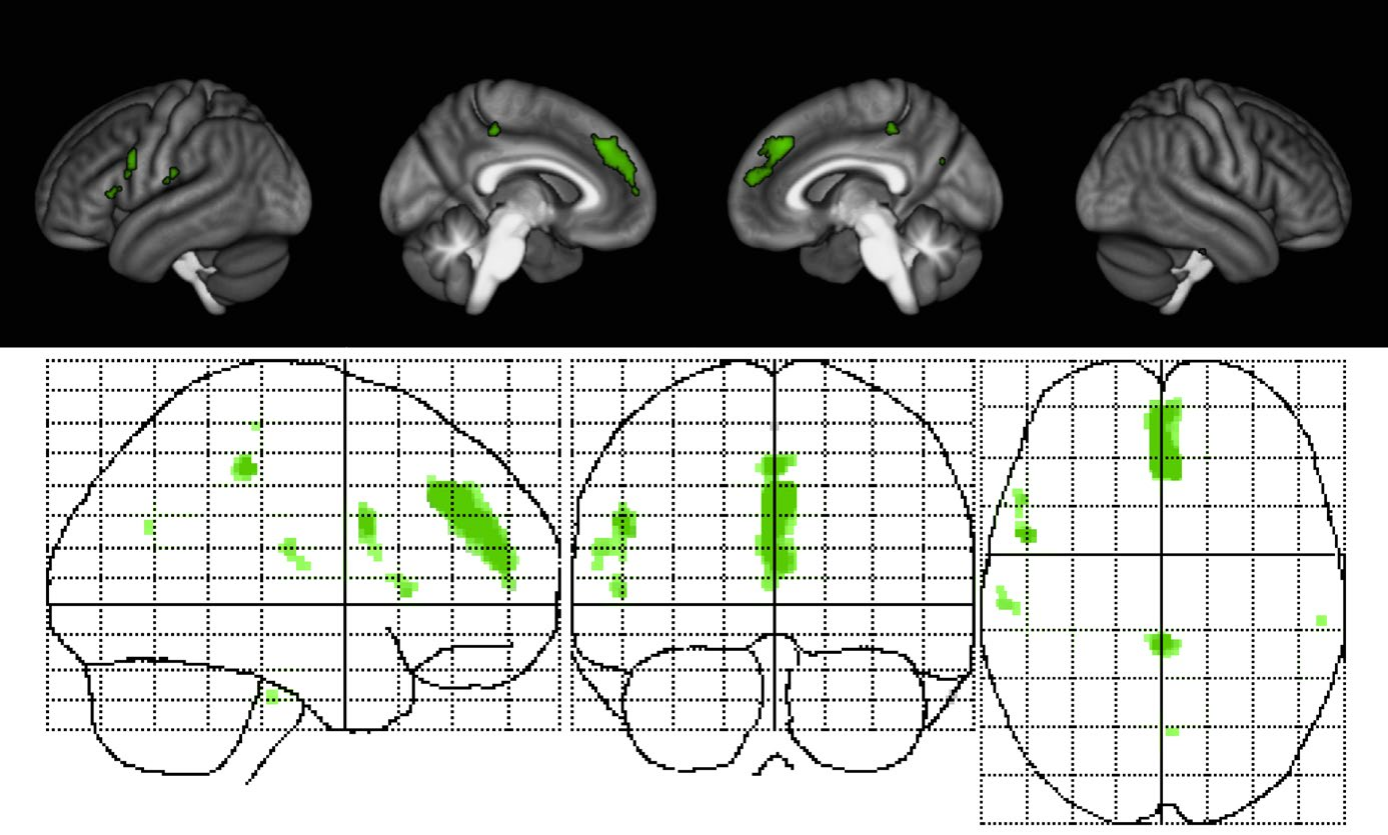
Overlap of the region of treatment‐induced sparing and the longitudinal cognitive disability‐specific atlas. Surface renderings (top) and maximum intensity projections (bottom) superimposed on the statistical parametric mapping standard glass brain

Finally, as we had observed a region of TIS on the one hand, and a DSA for the PASAT2 on the other, we next determined whether there was neuroanatomic overlap between these two regions. An overlap between the TIS and the DSA would support our hypothesis that the improvement in PASAT scores in the estriol + GA subjects was due to GM preservation in a clinically eloquent region. We observed that there was indeed overlap between the TIS and the DSA, a GM region within the volume preserved by estriol treatment that was also within the region where GM preservation correlated with improved performance on the PASAT2 (Figure 6).

## 4 DISCUSSION

Here, the effect of 12 months of estriol treatment on voxelwise GM atrophy in MS was studied for its relationship to clinical, as well as other imaging, outcomes. The volume of the TIS region correlated with performance on the PASAT, but not with the 9HPT or T25FW, suggesting that estriol treatment preferentially preserves GM associated with cognition. These results for localized clusters within the cerebral cortex here are consistent with previous findings demonstrating a correlation between whole cortical GM volume and PASAT improvement in the estriol‐treated, but not the placebo‐treated patients (Voskuhl et al., 2016).

A striking finding was that the overlap between the TIS and the cognitive DSA included a sizeable cluster that localized to the medial frontal cortex, a structure that has been implicated in problem solving (Bush, Luu, & Posner, 2000) and attention (Petersen & Posner, 2012; Posner & Petersen, 1990) and is known to be activated during arithmetic strategy selection (Taillan et al., 2015) and the counting Stroop test (Bush et al., 1998). In fact, one study reported that patients with focal injury (either infarction, hemorrhage, trauma, or resection of a benign tumor) in the medial frontal cortex had difficulty counting the numbers in a series of auditory stimuli (Shallice, Stuss, Alexander, Picton, & Derkzen, 2008). This biology‐driven result, which was not based on an a priori structure of interest, supports our hypothesis that atrophy of localized cortical GM clusters is directly associated with impairment of cognitive processing speed in MS patients.

Previous studies using VBM to analyze the effect of disease‐modifying drugs have reported GM atrophy of 1.25% per year after one year of treatment with natalizumab (Ciampi et al., 2017). Comparatively, we observed GM loss of 0.5% per year in the estriol + GA group compared to 1.5% per year in the placebo + GA group after one year of treatment. Interestingly, one study that followed 24 patients treated with natalizumab for 3 years reported no significant GM atrophy, although no placebo‐treated controls were evaluated (Mattioli et al., 2015). Small clusters of increased (143 and 76 voxels) and decreased (59 voxels) GM density were described in these patients. Clearly, natalizumab is an excellent anti‐inflammatory treatment that decreases relapses in MS. Therefore, we hypothesize that the relatively modest effect on localized GM sparing using VBM may be due to a lack of direct neuroprotective effects of natalizumab. Estrogens, in contrast, have been shown to be neuroprotective in a variety of neurologic disease models (Bode et al., 2008; Engler‐Chiurazzi, Brown, Povroznik, & Simpkins, 2017; Spence & Voskuhl, 2012; Suzuki, Brown, & Wise, 2009), including estriol treatment on cognitive electrophysiologic and neuropathologic outcomes in the MS model (Ziehn, Avedisian, Dervin, O'Dell, & Voskuhl, 2012).

Our data demonstrating preservation of cortical GM, particularly in the frontal cortex, are consistent with a recent report of a follow‐up study of estrogen therapy initiated within 3–36 months after the onset of menopause in healthy women (Kronos Early Estrogen Prevention Study (KEEPS) (Kantarci et al., 2018). Treatment with estradiol patch (Climara), but not oral conjugated equine estrogen (Premarin), reduced ageing‐related atrophy in the prefrontal cortex as compared to placebo treatment. This sparing of localized cortical GM in the estradiol‐treated group correlated with lower global cortical β‐amyloid deposition as measured by positron emission tomography (PET) imaging. However, no differences were observed between treatment groups on global cognitive function. Together, these data with ours suggest that specificity with respect to type and dose of estrogen treatment, as well as cognitive domain and neurodegenerative mechanism, are each important considerations in the effect of estrogen treatment on preventing brain atrophy and improving cognitive performance.

In this study, we observed that whole GM atrophy was decreased in estriol + GA‐treated compared to placebo + GA‐treated subjects at month 12 (*p* = 0.04), whereas previously this difference was not as robust (*p* = 0.1) (Voskuhl et al., 2016). This difference may be caused by the difference in approach (VBM versus pairwise Jacobian integration), differences in patient populations (111 subjects versus 133 subjects), or due to the rigorous, treatment‐blinded visual inspection for quality control that we performed on the imaging data.

There is ample evidence that there is a practice effect on PASAT performance (Tombaugh, 2006), so the parent trial used best practices developed for the MSFC (Solari, Radice, Manneschi, Motti, & Montanari, 2005) and had the subjects perform the PASAT three times prior to enrollment. Estriol + GA‐treated subjects exhibited an improvement in PASAT3 scores at month 12 compared to placebo + GA‐treated subjects. As the placebo + GA subjects underwent the PASAT at the same time points as estriol + GA subjects, this difference between groups cannot be due to a practice effect. This does not, however, preclude an improvement in learning ability in estriol + GA‐treated subjects in the setting of “practice.” Notably, the improvement in PASAT scores observed in all subjects was driven by improvement in the subgroup with impairment at baseline (PASAT3 scores less than 55), as no improvement occurred in those with little or no impairment at baseline (PASAT3 scores 55–60) (Voskuhl et al., 2016).

Determining the mechanism of treatments in MS patients is always challenging and requires insights from preclinical models. While the protective effect of estriol treatment on MS appears to be mediated in part by anti‐inflammatory mechanisms (Gold et al., 2009; Soldan, Alvarez Retuerto, Sicotte, & Voskuhl, 2003; Voskuhl & Gold, 2012), this is not mutually exclusive of direct neuroprotective effects, as these have been shown in the MS model (Crawford et al., 2010; Kim et al., 2018; MacKenzie‐Graham et al., 2012; Spence et al., 2011). Together, preclinical data in EAE have shown that estriol is acting to decrease microglial activation, induce remyelination, and increase synaptic plasticity (Kim et al., 2018; Ziehn et al., 2012). Further, we have shown that treatment with estrogens and estrogen receptor ligands prevented both cortical and cerebellar GM atrophy by MRI, which correlated with preserving axons, neurons, and synapses in EAE (Itoh et al., 2017; Kim et al., 2018; MacKenzie‐Graham et al., 2012). Consistent with this, treatment with estrogens has been associated with neuroprotection in mouse models of ischemic stroke, Alzheimer's disease, Parkinson's disease, and Huntington's disease (Bode et al., 2008; Morissette, Al Sweidi, Callier, & Di Paolo, 2008; Suzuki et al., 2009; Yue et al., 2005).

It is not known why the TIS was specifically spared by estriol treatment, as estrogen receptors are expressed widely in brain and not limited to these regions. We speculate that localized sparing may be a function of the level of GM pathology in specific regions, with half the RRMS patients in this trial having cognitive dysfunction. There was a much lower frequency of disabilities in walking and vision in this RRMS cohort. Perhaps, if more patients in our cohort had exhibited other disabilities, then other GM regions associated with those disabilities would have been spared by estriol treatment (e.g., primary motor cortex in patients with walking disability). This warrants further investigation.

Limitations of this study include the fact that the parent clinical trial was not powered for exploratory analyses of cognitive performance or GM atrophy. Nevertheless, we observed localized GM preservation in estriol + GA‐treated subjects, suggesting a robust effect of estriol treatment. Confirmation of these findings is needed in a phase 2 trial with cognitive testing as the primary outcome measure, a trial that is ongoing (clinicaltrials.gov trial identifier # NCT01466114). Secondarily, the imaging data for the trial were acquired across numerous performance sites and scanners, introducing some variability into the MRI measures and potentially decreasing our ability to detect additional biologic differences.

This approach, utilizing biologically defined regions of interest to evaluate the effects of neuroprotective therapies on GM changes, can be applied to other MS studies to localize GM preservation associated with improvement in other clinical disabilities beyond cognition. It may facilitate the development of disability‐specific biomarkers for use as outcome measures in neuroprotective treatment trials in MS. Further, this approach can be adapted for use in other neurodegenerative diseases, providing a potential outcome measure to assess the efficacy of neuroprotective treatments targeting each of several specific disabilities in these diseases (Itoh et al., 2018).

Early intervention with a neuroprotective agent, such as estriol, may help prevent the GM loss that is associated with cognitive impairment. Although our study is limited by the small sample size, that did not prevent us from uncovering statistically significant differences in localized GM sparing. Further investigation in a larger phase 3 trial is now warranted with cognition as a primary outcome and localized GM sparing as its biomarker.
